# A combination of midlife diabetes mellitus and the apolipoprotein E ε4 allele increase risk for cognitive decline

**DOI:** 10.3389/fnagi.2022.1065117

**Published:** 2022-11-17

**Authors:** Boung Chul Lee, Young Min Choe, Guk-Hee Suh, Ihn-Geun Choi, Jun Ho Lee, Hyun Soo Kim, Jaeuk Hwang, Dahyun Yi, Jee Wook Kim

**Affiliations:** ^1^Department of Psychiatry, Hallym University College of Medicine, Chuncheon, South Korea; ^2^Department of Neuropsychiatry, Hallym University Hangang Sacred Heart Hospital, Seoul, South Korea; ^3^Department of Neuropsychiatry, Hallym University Dongtan Sacred Heart Hospital, Hwaseong, South Korea; ^4^Department of Psychiatry, Seoul W Psychiatric Office, Seoul, South Korea; ^5^Department of Neuropsychiatry, Seoul National University Hospital, Seoul, South Korea; ^6^Department of Laboratory Medicine, Hallym University Dongtan Sacred Heart Hospital, Hwaseong, South Korea; ^7^Department of Psychiatry, Soonchunhyang University Seoul Hospital, Seoul, South Korea; ^8^Institute of Human Behavioral Medicine, Medical Research Center, Seoul National University, Seoul, South Korea

**Keywords:** midlife diabetes mellitus, APOE4, global cognitive decline, Alzheimer’s disease, diabetes mellitus

## Abstract

**Background:**

It has been suggested that diabetes mellitus (DM) and the apolipoprotein E (APOE) ε4 allele (APOE4) increase the risk for Alzheimer’s disease (AD) and cognitive decline. However, the evidence is sparse. We explored whether APOE4 status modulated the effects of midlife and late-life DM on global cognition of non-demented older adults.

**Methods:**

In all, 176 non-demented adults (age 65–90 years) were enrolled. All the participants underwent comprehensive clinical assessments including midlife and late-life DM evaluation and APOE genotyping. The global cognitive performance index was assessed by the total score (TS) of the Consortium to Establish a Registry for Alzheimer’s Disease neuropsychological battery.

**Results:**

We found a significant midlife DM × APOE4 interaction effect on the global cognitive performance. Subgroup analyses indicated that an association between midlife DM and decreased global cognitive performance was apparent only in older adults who were APOE4-positive, and not in those with APOE4-negative.

**Conclusion:**

Our findings from non-demented older adults suggest that midlife DM increases the risk for AD and cognitive decline, and this risk is modulated by APOE4 status. To prevent AD and cognitive decline, physicians should check for the possible coexistence of midlife DM and APOE4-positive status.

## Introduction

Diabetes mellitus (DM) has been reported to be a potential risk factor for cognitive impairment and dementia including Alzheimer’s disease (AD) (Koekkoek et al., 2015; Hu et al., 2020; Noguchi-Shinohara et al., 2022). Some human and preclinical studies have supported the idea that DM may cause cognitive impairment and dementia by impairing brain structure and metabolism, e.g., amyloid β (Aβ), tau, and glucose metabolism, as well as insulin signals and cerebrovascular dysfunction (Jayaraman and Pike, 2014; Gonzalez-Reyes et al., 2016; Shinohara and Sato, 2017; Sutherland et al., 2017; Beeri and Bendlin, 2020; Rasool et al., 2021). In particular, midlife or early-onset DM may hasten cognitive impairment and dementia in later life and could be more stronger associated with an increased risk of all dementia and AD dementia than late-onset DM (Mayor, 2014; Harvard Health Letter, 2015; Sadahiro et al., 2019; Wang and Liu, 2021).

Apolipoprotein E ε4 allele (APOE4) is the strongest risk gene for AD (Yamazaki et al., 2019) and affect the major pathway by which APOE4 increases the risk of AD by driving earlier and more abundant Aβ pathology in the brains of APOE4-positive older adults (Morris et al., 2010; Yamazaki et al., 2019). In addition, APOE4 may cause cognitive impairment and dementia by reducing functions in multiple brain homeostatic pathways, such as lipid transport, synaptic integrity and plasticity, glucose metabolism, and cerebrovascular function (Lowe et al., 2014; Yamazaki et al., 2019; Jeon et al., 2020).

Taken together, both major risk factors, i.e., DM and APOE4, may affect AD and cognitive dysfunction by sharing common pathways (Liu et al., 2013; Zhao et al., 2017; Shinohara et al., 2020). However, the interaction between DM and APOE4 as contributing to cognitive dysfunction and dementia has not yet been fully clarified. A cohort study showed a significant interaction between a DM history and the APOE genotype, in which any DM history affected the cognitive decline in APOE4-negative older adults, but not in APOE4-positive older adults (Shinohara et al., 2020). In contrast, a cross-sectional study on middle-aged to older adults revealed the modifying effect of the presence of APOE4 on the association between current DM and poor cognitive performance (Dore et al., 2009). Furthermore, a longitudinal study showed that APOE4 modifies the association between midlife DM and poor cognitive decline (Bangen et al., 2013). These conflicting results may be due to a difference in the DM period, i.e., midlife or late-life DM. Nevertheless, no study has yet investigated the interaction between DM and APOE4 in contributing to poor cognition and AD or dementia according to the presence of DM during the midlife and late-life period, respectively. Therefore, we examined the association between midlife and late-life DM and global cognition in non-demented older adults. We also explored the moderating effect of APOE4 on these associations. Additionally, we investigated the moderating effect of APOE4 on the associations between other vascular risks and global cognition for comparative purpose.

## Materials and methods

### Participants

This study is part of the General Lifestyle and AD (GLAD) study, which aimed to investigate how multi-faceted lifetime experiences and bodily changes contribute to the brain or cognitive changes related AD. Given that AD dementia stage may be too late to see the effects of disease-course modifying therapies or interventions due to irreversible neuronal loss (Sperling et al., 2011), early introduction of interventions before neuronal loss would be important for maximizing their beneficial effect. Moreover, the targets for developing disease-modifying therapies or interventions are shifting from patients with AD dementia to individuals in the predementia phase and even in the asymptomatic stage of AD (Epelbaum et al., 2017). In this context, the present study aimed to focus on non-demented older adults. As of March 2022, the study had enrolled 176 non-demented adults between 65 and 90 years of age: 63 cognitively normal (CN) adults and 113 adults with mild cognitive impairment (MCI). Participants were recruited from individuals who participated in a dementia screening program at the memory clinic of Hallym University Dongtan Sacred Heart Hospital, Hwaseong, South Korea. Those who volunteered were invited for an eligibility assessment. Volunteers from the community were recruited through recommendations from other participants, family members, friends, or acquaintances.

The CN group consisted of participants with a Clinical Dementia Rating (CDR) (Morris, 1993) score of 0 and no diagnosis of MCI or dementia. All participants with MCI met the current consensus criteria for amnestic MCI, including memory complaints confirmed by an informant, objective memory impairment; preservation of global cognitive function, independence in functional activities, and absence of dementia. Regarding objective memory impairment, the age-, education-, and sex-adjusted *z*-score was <−1.0 for at least one of the four episodic memory tests included in the Korean version of the Consortium to Establish a Registry for Alzheimer’s Disease (CERAD-K) neuropsychological battery: the word list memory, word list recall, word list recognition, and constructional recall tests (Morris et al., 1989; Lee et al., 2002, 2004). All individuals with MCI had a CDR score of 0.5. The exclusion criteria were the presence of a major psychiatric illness or a significant neurological or medical condition or comorbidity that could affect mental functioning, illiteracy, the presence of visual/hearing difficulties, and/or severe communication or behavioral problems that would make clinical examinations difficult, and use of an investigational drug. This study protocol was approved by the Institutional Review Board of the Hallym University Dongtan Sacred Heart Hospital and was conducted following the recommendations of the current version of the Declaration of Helsinki. The participants or their legal representatives gave informed consent.

### Clinical assessments

All participants underwent standardized clinical assessments by trained psychiatrists based on the GLAD study clinical assessment protocol, which incorporated the Korean version of the CERAD-K (Morris et al., 1989; Lee et al., 2002). Trained neuropsychologists administered the GLAD neuropsychological assessment protocol incorporating the CERAD-K neuropsychological battery (Lee et al., 2004) to all participants. The CERAD total score (TS), which was included in the CERAD-K neuropsychological battery (Lee et al., 2004), were selected as a measure of global cognitive function. A lower score indicates poorer cognitive performance (Lee et al., 2004). The TS was generated by summing the scores of the six tests in the CERAD neuropsychological battery including verbal fluency, the modified Boston naming test, word list memory, constructional praxis, word list recall, and word list recognition tests (Seo et al., 2010).

A vascular risk score (VRS) reflecting vascular risk burden was calculated using the number of vascular risk factors (DeCarli et al., 2004). All vascular risks (e.g., hypertension, DM, dyslipidemia, coronary heart disease, transient ischemic attack, and stroke) were assessed based on data collected by trained researchers during systematic interviews of participants and their family members. Reliable informants were interviewed to acquire accurate information.

Midlife and late-life vascular risks were deemed present if participants had been diagnosed in a clinic or were taking medications for vascular risk at the time from years 45 to 64 and 65 to the present, respectively.

### Measuring apolipoprotein E genotype

Apolipoprotein E was genotyped using the Seeplex ApoE ACE genotyping kit (Seegene, Seoul, South Korea). APOE4-positivity was defined as the presence of at least one ε4 allele.

### Statistical analysis

Multiple linear regression analyses were performed to examine the associations between midlife (or late-life DM) with and global cognition, multiple linear regression analyses were performed. The midlife (or late-life) DM was the independent variable in each analysis and TS was the dependent variable. We tested three models, controlling for the covariates in a stepwise manner. The first model did not include any covariates; the second model included age, sex, education, and the clinical diagnosis as covariates; and the third model included the covariates in the second model plus late-life VRS. Then, we tested the regression analyses including the midlife (or late-life) DM × APOE4-positivity interaction as an additional independent variable to test the hypothesis that APOE4-positivity modulates the association between midlife (or late-life) DM and the TS. When a significant midlife (or late-life) DM × APOE4-positivity interactive effect was found, we performed additional subgroup analyses using the regression model for the APOE4-negative and APOE4-positive groups separately. All statistical analyses were performed using SPSS Statistics software ver. 27 (IBM, Corp., Armonk, NY, USA).

## Results

### Participants

Table 1 summarizes the demographic and clinical characteristics of the participants. Of the 176 participants, 63 were CN and 113 were MCI. Supplementary Table 1 shows the demographic and clinical characteristics of the participants according to the midlife DM status. In total, 147 were DM-negative participants and 29 were DM-positive participants.

**TABLE 1:** Demographic and clinical characteristics of the older participants.

Characteristic	Overall
*n*	176
Age, years	72.99 (5.63)
Female, *n* (%)	122 (69.32)
Education, years	9.28 (4.53)
APOE4-positivity, *n* (%)	40 (22.73)
MCI, *n* (%)	113 (64.20)
MMSE	24.69 (3.60)
**Midlife vascular risks**	
DM	29 (16.48)
HTN	71 (40.34)
Dyslipidemia	43 (24.43)
Coronary heart disease	8 (4.55)
TIA	2 (1.14)
Stroke	2 (1.14)
**Late-life vascular risks**	
DM	43 (24.43)
HTN	104 (59.09)
Dyslipidemia	77 (43.75)
Coronary heart disease	27 (15.34)
TIA	0 (0.00)
Stroke	2 (1.14)
Midlife VRS, %	14.96 (17.10)
Current VRS, %	23.67 (18.38)
**Global cognitive performance**	
TS	58.66 (13.86)

APOE4, apolipoprotein E ε4 allele; MCI, mild cognitive impairment; MMSE, mini-mental state examination; DM, diabetes mellitus; HTN, hypertension; TIA, transient ischemic attack; VRS, vascular risk score; TS, total score of Consortium to Establish a Registry for Alzheimer’s Disease. Data are expressed as mean (SD), unless otherwise indicated.

### Association between diabetes mellitus, apolipoprotein E ε4 allele-positivity, and cognition

Midlife and late-life DM were not significantly associated with the TS (Supplementary Table 2). In addition, APOE4-positivity was not significantly associated with the TS (Supplementary Table 3).

### Moderating effect of apolipoprotein E ε4 allele-positivity on the association between diabetes mellitus and cognition

**FIGURE 1:**
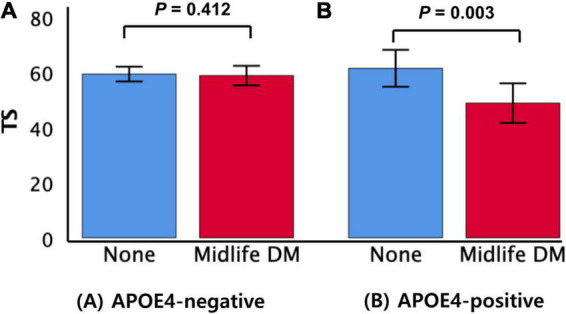
Box plots displaying midlife DM status and the TS in older participants by subgroup **(A)** APOE4-negative and **(B)** APOE4-positive. Error bars indicate standard errors. DM, diabetes mellitus; TS, total score of Consortium to Establish a Registry for Alzheimer’s Disease; APOE4, apolipoprotein E ε4 allele. Multiple linear regression analyses were performed after adjusting for potential confounders.

The midlife DM × APOE4-positivity interaction was significant in terms of the TS, indicating that APOE4-positivity moderated the association between the midlife DM and global cognitive impairment (Table 2). Further subgroup analyses showed that midlife DM was significantly associated with a low TS in the APOE4-positive but not in the APOE4-negative subgroup (Table 3 and Figures 1A,B). The interactions between other vascular risks and APOE-4 positivity were not significant (Table 2).

**TABLE 2:** The results of multiple linear regression analyses, including the interaction terms between vascular risks (DM, HTN, dyslipidemia, coronary heart disease, TIA, or stroke) in terms of predicting the TS.

	β	*P*
Midlife DM	0.079	0.230
APOE4-positivity	0.044	0.476
Midlife DM × APOE4-positivity	−0.203	0.007
Midlife HTN	−0.006	0.915
APOE4-positivity	0.005	0.941
Midlife HTN × APOE4-positivity	−0.071	0.379
Midlife dyslipidemia	0.119	0.059
APOE4-positivity	0.004	0.954
Midlife dyslipidemia × APOE4-positivity	−0.109	0.129
Midlife coronary heart disease	0.063	0.306
APOE4-positivity	−0.037	0.495
Midlife coronary heart disease × APOE4-positivity	−0.036	0.564
Midlife TIA	NA	NA
APOE4-positivity	NA	NA
Midlife TIA APOE4-positivity	NA	NA
Midlife stroke	NA	NA
APOE4-positivity	NA	NA
Midlife stroke APOE4-positivity	NA	NA
Late-life DM	0.041	0.524
APOE4-positivity	−0.003	0.964
Late-life DM × APOE4-positivity	−0.085	0.255
Late-life HTN	−0.038	0.531
APOE4-positivity	<0.001	0.998
Late-life HTN × APOE4-positivity	−0.050	0.616
Late-life dyslipidemia	0.066	0.274
APOE4-positivity	−0.042	0.581
Late-life dyslipidemia × APOE4-positivity	−0.012	0.891
Late-life coronary heart disease	0.080	0.176
APOE4-positivity	−0.029	0.623
Late-life coronary heart disease × APOE4-positivity	−0.034	0.593
Late-life TIA	NA	NA
APOE4-positivity	NA	NA
Late-life TIA APOE4-positivity	NA	NA
Late-life stroke	NA	NA
APOE4-positivity	NA	NA
Late-life stroke APOE4-positivity	NA	NA

DM, diabetes mellitus; HTN, hypertension; TIA, transient ischemic attack; TS, total score of Consortium to Establish a Registry for Alzheimer’s Disease; APOE4, apolipoprotein ε4 allele; VRS, vascular risk score; NA, not assessable. Multiple linear regression model included vascular risks, APOE4-positivity, and the interaction between vascular risks and APOE4-positivity treated as the independent variables; for potential confound factors (age, sex, education, and clinical diagnosis) were treated as covariates; and TS treated as the dependent variable.

**TABLE 3:** The results of the multiple linear regression analyses for the associations between midlife DM and the TS by the APOE4-positivity subgroup.

	APOE4-negative,		APOE4-positive,	
	*n* = 136		*n* = 40	
	β	*P*	β	*P*
**Midlife DM**				
Model 1^a^	0.048	0.581	−0.413	0.008
Model 2^b^	0.068	0.245	−0.353	0.007
Model 3^c^	0.054	0.412	−0.445	0.003

DM, diabetes mellitus; TS, total score of Consortium to Establish a Registry for Alzheimer’s Disease; APOE4, apolipoprotein E ε4 allele; VRS, vascular risk score. ^a^Unadjusted. ^b^Adjusted for age, sex, education, and clinical diagnosis. ^c^Adjusted for age, sex, education, clinical diagnosis, and current VRS.

## Discussion

This study of older adults found that midlife DM worsened global cognition, i.e., the TS, which was moderated by the effect of APOE4 on the association between midlife DM and decreased global cognition. Previous studies have suggested that DM and APOE4 are major risk factors for AD and related cognitive decline, and there are many reports that each has a significant association with cognitive decline (Morris et al., 2010; Liu et al., 2013; Jayaraman and Pike, 2014; Lowe et al., 2014; Koekkoek et al., 2015; Gonzalez-Reyes et al., 2016; Shinohara and Sato, 2017; Sutherland et al., 2017; Zhao et al., 2017; Yamazaki et al., 2019; Beeri and Bendlin, 2020; Hu et al., 2020; Jeon et al., 2020; Shinohara et al., 2020; Rasool et al., 2021; Noguchi-Shinohara et al., 2022). The coexistence of these two risk factors is thought to have a more devastating effect on AD and related cognitive decline, but few studies have examined the interaction between the two. In addition, midlife and late-life DM may have different effects on cognitive function (Mayor, 2014; Harvard Health Letter, 2015; Sadahiro et al., 2019; Wang and Liu, 2021) in addition to the moderation effect of APOE4. Nevertheless, no study has examined DM, APOE4, and the DM interaction by dividing the exposure to DM into middle age and older age, respectively.

This is the first study to demonstrate that APOE4 has a significant moderating effect on the association between DM and cognitive decline in middle age, but not in older age. This may be because participants with midlife DM have a longer duration of exposure to DM and are more likely to exacerbate the progression than those with late-life DM, including late-onset DM. These findings are consistent with the results of previous studies regarding the stronger effect of early-onset DM on the risk of AD dementia and cognition than late-onset DM (Mayor, 2014; Harvard Health Letter, 2015; Sadahiro et al., 2019; Wang and Liu, 2021). Moreover, this midlife DM is likely to have an interacting or synergistic effect in APOE4-positive older adults who may be susceptible to AD or related cognitive decline. Contrary to the results of our study, a longitudinal study demonstrated that non-demented older adults aged 65 years and over who have late-life DM and APOE4 are at a substantially higher risk of cognitive decline (Haan et al., 1999). Although their findings did not confirm the presence or absence of DM in the middle age, it seems similar to our findings on the DM-APOE4 interaction for cognitive decline when the proportion of DM that starts in middle age, i.e., midlife DM, is high among older adults with late-life DM. A cross-sectional study with middle to older adults showed a modifying effect of the presence of APOE4 on the association between current DM and poor cognitive performance (Dore et al., 2009). Their study included a significant number of middle-aged as well as older adults, so it is likely that their findings were similar to ours due to the significant proportion of middle-aged onset DM. Taken together, these conflicting results may be due to differences in the DM exposure period, i.e., midlife and late-life DM, as well as the proportion of participants with midlife DM.

In the present study, this moderating effect was only seen in the APOE4-positive group, and not in the APOE4-negative group. The majority of the findings of previous studies support our results (Haan et al., 1999; Dore et al., 2009; Bangen et al., 2013). However, a cohort study suggested that this moderating effect appeared in the APOE4-negative group, and commented that such effects may be masked by the presence of APOE4, which itself increases the risk of vascular problems (Shinohara et al., 2020). The difference may be due to the DM exposure period and the accuracy of cognitive assessment scales. Unlike our study, they defined the presence of DM as any DM history and used relatively simple scales, such as the CDR or mini-mental state examination (MMSE). In contrast, we defined DM as midlife or late-life DM and used the TS of the CERAD, which has much broader cognitive domains and scores with a wider range than CDR or MMSE, to reflect minimal changes in cognition over the global domains. Other possibilities include differences in the ratio and combination of APOE2 and 3 alleles in the APOE4-negative group and differences in clinical expression according to race (Barnes and Bennett, 2015).

The precise mechanism by which midlife DM and APOE4 interact to increase AD and cognitive dysfunction is unclear. As one of the most probable hypotheses, that accumulation of advanced glycation end products (Vistoli et al., 2013), a biomarker implicated in the development and progression of oxidative-based diseases such as DM (Yamagishi et al., 2012) and AD (Sasaki et al., 1998; Grillo and Colombatto, 2008; Li et al., 2012), may play a role in neurodegeneration given the co-localization of advanced glycation end products in the Aβ and tau pathology in AD (Sasaki et al., 1998; Grillo and Colombatto, 2008; Li et al., 2012). In addition, APOE4 is associated with a threefold greater advanced glycation end products-binding activity compared to the APOE3 isoform indicating that advanced glycation end products caused by APOE4 interactions may contribute to the formation of Aβ and tau deposition (Li and Dickson, 1997). As other mechanisms, insulin-degrading enzyme (Kurochkin et al., 2018), insulin resistance (Sedzikowska and Szablewski, 2021), and acylated glucagon-like peptide 1 (GLP-1) analog have been proposed to link DM and AD, and the role of APOE4 between them is being investigated. In particular, liraglutide, a novel DM drug with a GLP-1 agonist action, is currently undergoing a clinical trial, i.e., the ELAD trial (Evaluating Liraglutide in Alzheimer’s Disease NCT01843075), and is being investigated for its benefits on changes in AD biomarkers and clinical measures in addition to age and APOE4 status (Femminella et al., 2020).

In this study, no interactions were detected between other vascular risks and APOE4 on global cognitive decline. A few studies have been published on the possible moderating effect of APOE4 on the association between other vascular risks [such as hypertension (Yasuno et al., 2012), atherosclerosis (Haan et al., 1999), and peripheral vascular disease (Haan et al., 1999)] and cognitive decline. As these vascular risks have the potential to interact with each other, systematic follow-up and repeat studies are needed to clarify the relationship.

## Strengths and limitations

To the best of our knowledge, this is the first study to show a moderating effect of APOE4 on the association between midlife DM and global cognitive decline. The present findings did not change even after controlling for potential confounders and late-life vascular risks. Our study had some limitations. First, as this was a cross-sectional study, causal relationships could not be inferred. Long-term prospective studies are needed. In addition, the small number of participants decreased statistical power, thus, contributing to the null result for the relationship between midlife DM, APOE4-positivity, and global cognition. A study with a larger sample size is required. Second, about 64% of the study participants were diagnosed with MCI, which may also raise some concerns about the accuracy of self-report for midlife-DM status. However, although participants with MCI have problems for their recent memory, their remote memory is very well-preserved (Leyhe et al., 2009). Therefore, it is not likely that participants with MCI reported their history for midlife DM status history more erroneously because the self-report for midlife DM status mainly depends on remote memory rather than recent memory. In addition, even when we controlled for the clinical diagnosis (CN vs. MCI) as an additional covariate in Model 3 (Tables 2, 3 and Supplementary Tables 2, 3), the results were still very similar. Additionally, the accuracy of the information of DM was verified by reliable informants or by reviewing available medical charts. Third, we did not statistically analyze the duration, severity, complications, or types of DM. However, we analyzed the interaction of DM with APOE4 on global cognition in middle age and older age, i.e., midlife DM and late-life DM, based on the evidence of two significant lifetime periods vulnerable to AD and related cognitive decline (Mayor, 2014; Harvard Health Letter, 2015; Koekkoek et al., 2015; Gottesman et al., 2017; Sadahiro et al., 2019; Hu et al., 2020; Wang and Liu, 2021; Noguchi-Shinohara et al., 2022). Nevertheless, there remains a possibility that treatment response (e.g., well-controlled DM or not) for presence and severity of hyperglycemia may affect our findings. We assessed fasting blood glucose levels, which may be considered as a biomarker for the presence and severity of hyperglycemia, and obtained the same results after controlled the fasting glucose level as an additional covariate (Supplementary Tables 4, 5). Lastly, we could not statistically analyze the dose effect of APOE4 for the moderating effect on DM and global cognition because only one subject with E4E4 was detected among all of the participants.

## Conclusion

Our findings suggest that midlife DM increases the risk for AD and cognitive decline, and this risk is modulated by APOE4 status. To prevent AD and cognitive decline, physicians should check for the possible coexistence of midlife DM and APOE4-positive status.

## Data availability statement

The study data are not freely accessible because the IRB of the Hallym University Dongtan Sacred Heart Hospital prevents public sharing of such data for privacy reasons. However, the data are available on reasonable request after IRB approval. Requests for data access can be submitted to an independent administrative coordinator by e-mail (yoon4645@gmail.com).

## Ethics statement

The studies involving human participants were reviewed and approved by the Institutional Review Board of the Hallym University Dongtan Sacred Heart Hospital. The patients/participants provided their written informed consent to participate in this study.

## Author contributions

JK conceived and designed the study, served as principal investigator, and supervised the study. BL, YC, G-HS, I-GC, JL, HK, JH, DY, and JK were involved in acquisition, or analysis and interpretation of the data and helped to draft the manuscript. JK, BL, YC, G-HS, I-GC, JL, JH, HK, and DY were major contributors in writing the manuscript and critically revising the manuscript for intellectual content. All authors read and approved the final manuscript.
