# Supplementary material for: A combination of midlife diabetes mellitus and the apolipoprotein E ε4 allele increase risk for cognitive decline
Source: Front Aging Neurosci. 2022 Nov 17;14:1065117. doi: 10.3389/fnagi.2022.1065117 (PMC9715424; doi:10.3389/fnagi.2022.1065117)
Supplement: Supplementary file 1 [file Data_Sheet_1.pdf]

Supplementary Materials

Table S1. Demographic and clinical characteristics of older participants according to midlife DM

Table S2. The results of the multiple linear regression analyses for the associations between midlife DM or late-life DM and the TS

Table S3. The results of the multiple linear regression analyses for the associations between APOE4-positivity and the TS

Table S4. The results of multiple linear regression analyses, including the interaction terms between DM in terms of predicting the TS

Table S5. The results of the multiple linear regression analyses for the associations between midlife DM and the TS by the APOE4-positivity subgroup

This supplemental material has been provided by the authors to give readers additional information about their work.

Table S1. Demographic and clinical characteristics of older participants according to midlife DM

Characteristic	Midlife DM status		*P*
	DM-negative	DM-positive	
n	147	29	
Age, y	72.73 (5.79)	74.31 (4.58)	0.167 [a]
Female, n (%)	102 (69.39)	20 (68.97)	0.964 [b]
Education, y	9.28 (4.58)	9.28 (4.30)	0.997 [a]
APOE4-positivity, n (%)	28 (19.05)	12 (41.38)	0.009 [b]
MCI, n (%)	93 (63.27)	20 (68.97)	0.558 [b]
MMSE	24.87 (3.46)	23.76 (4.21)	0.129 [a]
Midlife vascular risks			
DM	0 (0.00)	29 (100.00)	<0.001 [b]
HTN	48 (32.65)	23 (79.31)	<0.001 [b]
Dyslipidemia	32 (21.77)	11 (37.93)	0.064 [b]
Coronary heart disease	7 (4.76)	1 (3.45)	1.000 [c]
TIA	1 (0.68)	1 (3.45)	0.305 [c]
Stroke	0 (0.00)	2 (6.90)	1.000 [c]
Late-life vascular risks			
DM	16 (10.88)	27 (93.10)	<0.001 [b]
HTN	80 (147)	24 (82.76)	0.005 [b]
Dyslipidemia	62 (42.18)	15 (51.72)	0.897 [b]
Coronary heart disease	19 (12.93)	8 (27.59)	0.086 [c]
TIA	0 (0.00)	0 (0.00)	
Stroke	0 (0.00)	2 (6.90)	1.000 [c]
Midlife vascular risk score, %	10.32 (13.42)	38.51 (14.16)	<0.001 [a]
Current vascular risk score, %	20.18 (16.81)	41.38 (15.82)	<0.001 [a]
Global cognitive performance			
TS	59.16 (13.75)	56.14 (14.43)	0.284 [a]

Abbreviations: APOE4, apolipoprotein E ε4 allele; MCI, mild cognitive impairment; MMSE, mini-mental state examination; DM, diabetes mellitus; HTN, hypertension; TIA, transient ischemic attack; VRS vascular risk score; TS total score of Consortium to Establish a Registry for Alzheimer's Disease.

Data are expressed as mean (standard deviation), unless otherwise indicated.

[a] by one-way analysis of variance.

[b] by chi-square test.

[c] by fisher exact test.

Table S2. The results of the multiple linear regression analyses for the associations between midlife DM or late-life DM and the TS

	Midlife DM		Late-life DM	
	β	*P*	*β*	*P*
Dependent variable: TS				
Model 1 [a]	-0.081	0.284	-0.101	0.182
Model 2 [b]	-0.035	0.511	-0.006	0.911
Model 3 [c]	-0.058	0.321	-0.042	0.544

Abbreviations: DM diabetes mellitus; APOE4 apolipoprotein E ε4 allele; TS total score of Consortium to Establish a Registry for Alzheimer's Disease; VRS vascular risk score.

[a] Unadjusted.

[b] Adjusted for age, sex, education. and clinical diagnosis.

[c] Adjusted for age, sex, education, clinical diagnosis, and late-life VRS.

Table S3. The results of the multiple linear regression analyses for the associations between APOE4-positivity and the TS

	APOE4-positivity	
	β	*P*
Dependent variable: TS		
Model 1 [a]	-0.115	0.127
Model 2 [b]	-0.043	0.424
Model 3 [c]	-0.047	0.383

Abbreviations: DM diabetes mellitus; APOE4 apolipoprotein E ε4 allele; TS total score of Consortium to Establish a Registry for Alzheimer's Disease; VRS vascular risk score.

[a] Unadjusted.

[b] Adjusted for age, sex, education. and clinical diagnosis.

[c] Adjusted for age, sex, education, clinical diagnosis, and late-life VRS.

Table S4. The results of multiple linear regression analyses, including the interaction terms between DM in terms of predicting the TS

	β	*P*
Midlife DM	0.070	0.331
APOE4-positivity	0.046	0.440
Midlife DM × APOE4-positivity	-0.185	0.007
Late-life DM	0.041	0.547
APOE4-positivity	0.006	0.930
Late-life DM × APOE4-positivity	-0.068	0.333

Abbreviations: DM diabetes mellitus; TS total score of Consortium to Establish a Registry for Alzheimer's Disease; APOE4 apolipoprotein ε4 allele.

Multiple linear regression model included DM, APOE4-positivity, and the interaction between DM and APOE4-positivity treated as the independent variables; for potential confound factors (age, sex, education, clinical diagnosis, and fasting glucose) were treated as covariates; and TS treated as the dependent variable.

Table S5. The results of the multiple linear regression analyses for the associations between midlife DM and the TS by the APOE4-positivity subgroup

	APOE4-negative, n = 136		APOE4-positive, n = 40	
	β	*P*	*β*	*P*
Midlife DM				
Model 1 [a]	0.048	0.581	-0.413	0.008
Model 2 [b]	0.068	0.245	-0.353	0.007
Model 3 [c]	0.054	0.412	-0.445	0.003
Model 4 [d]	0.085	0.253	-0.540	0.001

Abbreviations: DM diabetes mellitus; TS total score of Consortium to Establish a Registry for Alzheimer's Disease; APOE4 apolipoprotein E ε4 allele; VRS vascular risk score.
[a] Unadjusted.
[b] Adjusted for age, sex, education. and clinical diagnosis.
[c] Adjusted for age, sex, education, clinical diagnosis, and current VRS.
[d] Adjusted for age, sex, education, clinical diagnosis, current VRS, and fasting glucose.
